# Overbias Photon Emission from Light-Emitting Devices Based on Monolayer Transition Metal Dichalcogenides

**DOI:** 10.1021/acs.nanolett.3c03155

**Published:** 2023-12-04

**Authors:** Shengyu Shan, Jing Huang, Sotirios Papadopoulos, Ronja Khelifa, Takashi Taniguchi, Kenji Watanabe, Lujun Wang, Lukas Novotny

**Affiliations:** † Photonics Laboratory, ETH Zürich, 8093 Zürich, Switzerland; ‡ International Center for Materials Nanoarchitectonics, National Institute for Materials Science, 1-1 Namiki, Tsukuba 305-0044, Japan; § Research Center for Functional Materials, National Institute for Materials Science, 1-1 Namiki, Tsukuba 305-0044, Japan

**Keywords:** transition metal dichalcogenides, van der Waals LED, overbias photon emission, exciton generation, multielectron tunneling, energy transfer

## Abstract

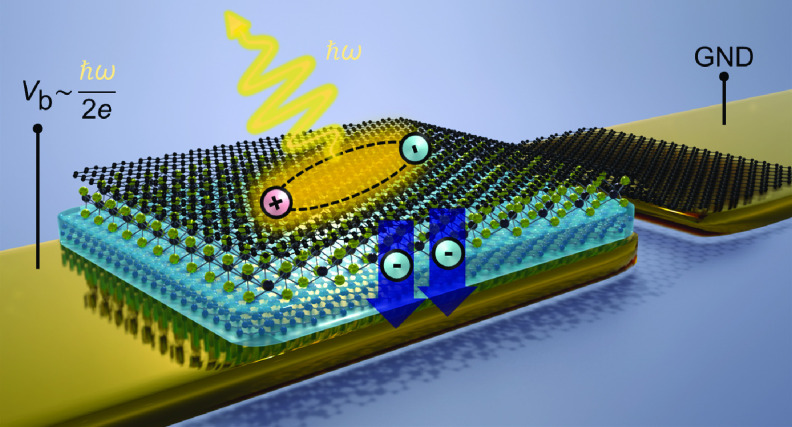

Tunneling light-emitting devices (LEDs) based on transition metal dichalcogenides (TMDs) and other two-dimensional (2D) materials are a new platform for on-chip optoelectronic integration. Some of the physical processes underlying this LED architecture are not fully understood, especially the emission at photon energies higher than the applied electrostatic potential, so-called overbias emission. Here we report overbias emission for potentials that are near half of the optical bandgap energy in TMD-based tunneling LEDs. We show that this emission is not thermal in nature but consistent with exciton generation via a two-electron coherent tunneling process.

In 2015, the first two-dimensional (2D) material-based tunneling light-emitting device (LED) was realized.^1,2^ It employed graphene (Gr) as a conductor for electrical contacts, transition metal dichalcogenides (TMDs) as semiconductors, and hexagonal boron nitride (hBN) as an insulator. This LED architecture has inspired investigations on cavity integration,^3,4^ single defect LEDs,^5^ and exciton modulation.^6^ It also opened up a new perspective for integrated on-chip optoelectronic devices.^7^

A typical device architecture is shown in Figure 1a. It consists of a Gr-hBN-WSe_2_-hBN-Gr heterostructure with two monolayer Gr flakes acting as transparent electrodes and two hBN multilayers defining the tunnel barriers. A monolayer of WSe_2_ is sandwiched in the middle and serves as the active material. Such double-tunnel barrier LEDs provide large-area exciton light emission with an external quantum efficiency (EQE) on the order of 10^–2^ at room temperature.^1,2^ Here, excitons are formed by the charge injection of both electrons and holes into the active layer. This requires the applied bias potential (*eV*_b_, where *e* is the elementary charge and *V*_b_ is the bias voltage) to be larger than the optical bandgap energy so that electrons and holes can tunnel from the Gr electrodes to WSe_2_, thereby forming excitons.^8^

**Figure 1:**
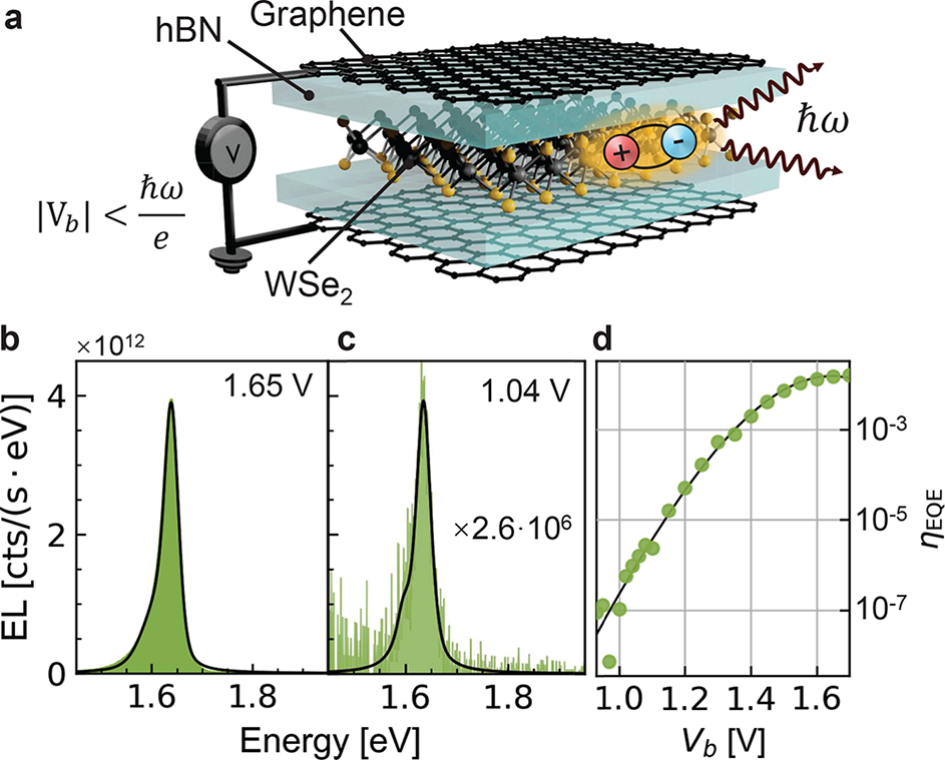
(a) Illustration of a double-barrier tunneling LED. The junction is encapsulated in hBN on both sides (not shown). (b, c) EL spectra of the double-barrier LED for *V*_b_ = 1.65 V and *V*_b_ = 1.04 V, respectively. The measured spectra (green areas) are fitted with the sum of two pseudo-Voigt functions (black lines) representing the A-exciton and trion. (d) EQE (in the spectral range from 1.4 to 1.8 eV) as a function of applied bias. The green dots represent data points, and the black curve is a guide to the eye.

However, there are also alternative ways to generate excitons for light emission such as by energy transfer. This process involves inelastic electron tunneling (IET), in which the electron couples its energy to TMD excitons during the tunneling process.^9−11^ Such energy transfer can occur efficiently in van der Waals (vdW) heterostructures and is due to strong near-field coupling between the tunneling electrons and the active material. Thus, excitons in TMDs can be generated either by charge injection or by energy transfer. In both processes energy conservation requires that the bias potential *eV*_b_ is larger than the optical bandgap energy *ℏ*ω_BG_ (*ℏ*ω_BG_ ≃ 1.64 eV for monolayer WSe_2_ at room temperature,^12^ where *ℏ* is the reduced Planck constant and ω_BG_ is the angular transition frequency); no excitonic photon emission is expected for *eV*_b_ < *ℏ*ω_BG_.^8,9^

In this paper, we report on exciton light emission from a monolayer TMD tunneling LED driven by bias potentials (*eV*_b_ ≃ 1.00 eV) much smaller than the optical bandgap energy (*ℏ*ω_BG_ ≃ 1.64 eV). To identify the physical origins of this overbias emission, we perform electroluminescence (EL) measurements on various LED designs and at different temperatures.

In addition to double-barrier LEDs we also investigate single-barrier Gr-TMD-hBN-gold heterostructures with TMD = {WSe_2_, MoSe_2_}. Compared with double-barrier LEDs, single-barrier LEDs can reach higher currents under the same bias voltage, thus allowing us to observe exciton emission at very low bias voltages. With this architecture, we start to detect light emission from the A-exciton in WSe_2_ at 0.81 V and at 0.74 V in MoSe_2_. The measured threshold voltages correspond to approximately half the optical bandgap energies. This observation hints at a second-order energy transfer process based on multielectron tunneling.^13−15^

We note that overbias emission has been observed before in light-emitting junctions, apart from vdW heterostructures. Depending on experimental conditions, this emission can be generated by thermal upconversion,^16,17^ non-thermal equilibrium carrier generation,^18−21^ and coherent multielectron processes.^13−15,22,23^ Also, upconversion in 2D materials has been accomplished optically via two-photon excitation^24^ or facilitated by an intermediate state, for example, by Auger scattering of interlayer excitons.^25^ Nevertheless, electrically driven overbias emission has never been reported in monolayer TMD-based LEDs.

We first describe our results for the double-barrier LED shown in Figure 1a. The core structure is a vertical assembly of Gr-hBN-WSe_2_-hBN-Gr, in which two Gr flakes serve as electrodes. The hBN thickness corresponds to 4 ± 1 atomic layers. This tunnel junction is encapsulated in two thick hBN flakes. The full encapsulation creates a homogeneous dielectric environment, enhancing uniformity of both the electrical properties of graphene^26^ and the optical properties of TMDs.^27^ We fabricate our devices by using the dry pick-up and transfer method,^28^ where we transfer the entire device onto a glass coverslip. After transfer we fabricate edge contacts to the two graphene electrodes.^29,30^ EL is collected with an oil-immersion objective from the glass side and detected by a spectrometer (see the Supporting Information, Sections I and II).

Monolayer WSe_2_ has an electronic bandgap of ∼1.82 eV^31^ and an optical bandgap of ∼1.64 eV at room temperature.^12^ Based on energy conservation, we expect that electrical generation of excitons requires bias potentials *eV*_b_ that are larger than the optical bandgap energy.^8^ In order to generate excitons at a bias below this threshold, higher-order processes or phonon-assisted interactions are required. Figure 1b shows a representative EL spectrum for *V*_b_ = 1.65 V. The peak of the spectrum centers at ∼1.64 eV, which corresponds to the A-exciton of WSe_2_.^8^ The asymmetric broadening at lower energies can be associated with trions.^8^ However, we also observe exciton light emission for *eV*_b_ significantly smaller than the optical bandgap. As an example, Figure 1c shows the EL spectrum for *V*_b_ = 1.04 V. Compared to Figure 1b, this spectrum has the same main peak position and similar line width, indicating that the spectrum is also dominated by the contribution from A-excitons. The spectral shape remains similar, but the intensity and hence the EQE decrease. We define EQE as , where Γ^X^ is the photon count rate in the spectral range from 1.4 to 1.8 eV and *I* is the electrical current (for more details, see the Supporting Information, Section III). As shown in Figure 1d, the EQE drops exponentially with decreasing *V*_b_ and disappears in the noise floor at ∼0.93 V. To extend the measurement range to even lower bias voltages, we require a higher emission intensity and hence a higher tunnel current. Therefore, in a next step, we eliminate one of the tunnel barriers and repeat the measurements for a single-barrier device.

The architecture of a single-barrier LED is shown in Figure 2a. The device is composed of a Gr-WSe_2_-hBN-gold heterostructure, where the monolayer Gr is in contact with a second gold electrode. As shown in Figure 2c, by using a single-barrier device (hBN with 3 ± 1 atomic layers), we are able to increase the current density by ∼4 orders of magnitude over the previous double-barrier device.

**Figure 2:**
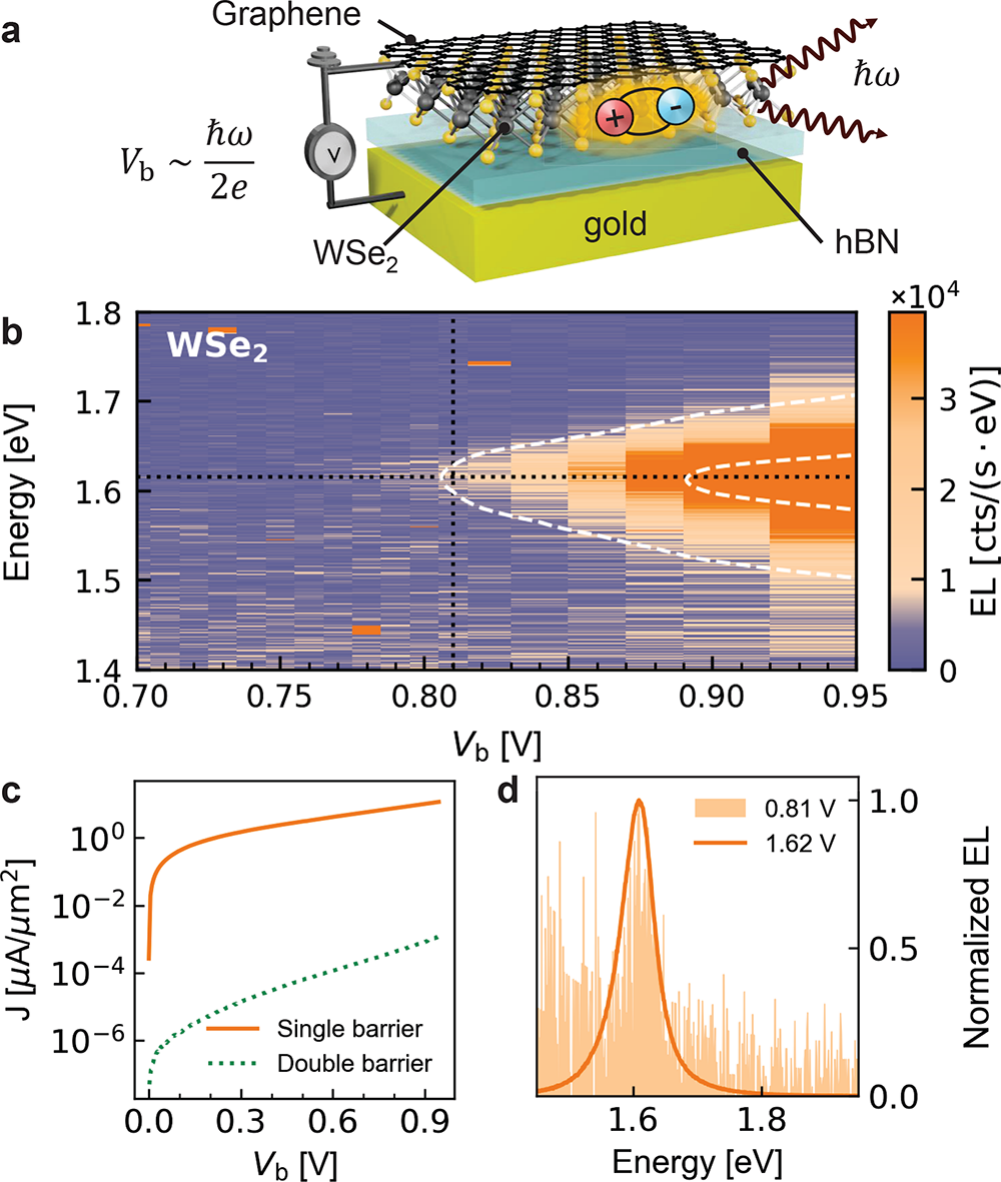
(a) Illustration of a single-barrier LED. The stack is encapsulated by a top hBN flake (not shown). (b) EL spectra for *V*_b_ ranging from 0.7 to 0.95 V. The horizontal dotted line indicates the neutral exciton energy (1.62 eV), and the vertical dotted line denotes the threshold for exciton emission. There is a factor of 10 difference between the two dashed contour lines (see Section V of the Supporting Information for more spectra). (c) Current density–voltage (*J*–*V*) curve of a single- and double-barrier LED. (d) Normalized EL spectra for *V*_b_ = 0.81 and 1.62 V.

A typical EL spectrum of the single-barrier device obtained at 1.62 V is shown in Figure 2d. The spectrum has a peak at ∼1.62 eV, which is slightly red-shifted compared to that of the double-barrier LED. Consequentially, we assign this peak to the neutral A-exciton, which is shifted to lower energies due to the stronger dielectric screening of the directly contacting Gr.^32,33^ The overall EL intensity is moderately quenched compared to the double-barrier device, and the spectrum becomes trion-free due to both charge and energy transfer.^34,35^Figure 2b shows the EL spectra as a function of *V*_b_ in the overbias emission regime. The horizontal axis represents the bias voltage with each vertical cross section denoting an EL spectrum corresponding to the applied bias. As we gradually lower *V*_b_, the exciton peak remains visible in the spectrum, even for *eV*_b_ = 0.81 eV (vertical dotted line), corresponding to half of the WSe_2_ optical bandgap energy (*ℏ*ω_BG_ = 1.62 eV). The EL spectrum for *V*_b_ = 0.81 V is shown in Figure 2d (light orange area). Its shape is almost identical with that of the spectrum recorded for 1.62 V (solid orange curve). This observation hints at a second-order process involving two electrons.

In order to further strengthen our interpretation, we replace WSe_2_ by MoSe_2_, which has a lower bandgap and should therefore lead to EL at even lower bias voltages. Furthermore, it is known that the exciton emission from MoSe_2_ is less affected by Gr quenching,^34^ thus yielding stronger EL emission and providing a better signal-to-noise ratio. Figure 3a shows voltage-dependent EL spectra of a MoSe_2_-based device. When the bias voltage V_b_ is 0.70 V, the spectrum is primarily characterized by background noise. As the bias is increased, the first feature, near 1.56 eV (horizontal dotted line), appears at 0.74 V (vertical dotted line). This feature is associated with the red-shifted A-exciton (1s state) of monolayer MoSe_2_.^34^ The threshold bias potential of 0.74 eV is again much lower than the photon energy of 1.56 eV. At higher biases, two side peaks appear near 1.66 and 1.74 eV. According to their energy offsets relative to the A-exciton, we assign the first to the 2s and 3s states of A-exciton and the second to the B-exciton.^36,37^ We estimate the binding energy of the A-exciton in our device to be ∼133 meV by the energy difference between the ground state and excited states. This value is smaller than those reported in refs (34) and (36), and we attribute this to the dielectric screening from both Gr and gold electrodes.

**Figure 3:**
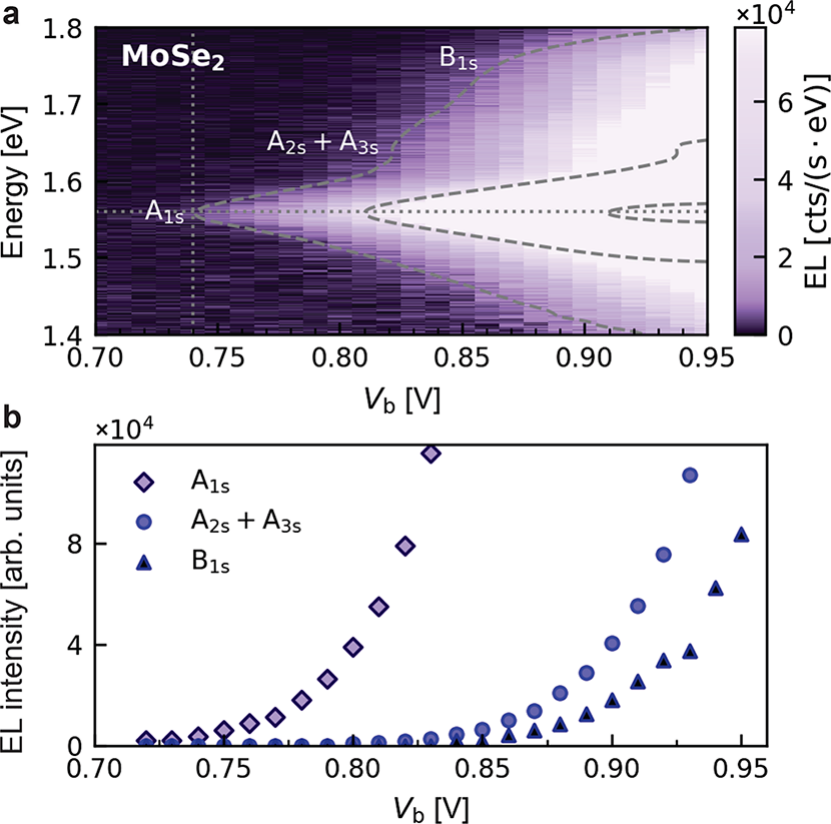
(a) EL spectra of a single-barrier LED based on MoSe_2_. The horizontal dotted line indicates the neutral exciton energy (1.56 eV), and the vertical dotted line denotes the threshold for exciton emission. There is a factor of 10 between adjacent dashed contour lines. (b) Dependence of the integrated EL intensity of A_1s_, A_2s_ + A_3s_, and B_1s_ excitons on bias voltage.

To analyze the voltage dependence of these three features, we fit the spectra with three pseudo-Voigt functions. The corresponding fitting amplitudes are plotted in Figure 3b as a function of the bias voltage (see the Supporting Information, Section IV). We observe that the three peaks emerge at different bias voltages: the lowest state of A-exciton with a peak position near 1.56 eV appears for *V*_b_ > 0.74 V, the 2s and 3s excited states near 1.66 eV have an onset voltage of 0.82 V, and the B-exciton with the highest energy (∼1.74 eV) emerges near *V*_b_ = 0.86 V. Altogether, each of the three features in MoSe_2_ emerges near bias potentials of half the photon energy (e*V*_b_ ≃ *ℏ*ω/2), similar to the WSe_2_ device.

Besides a second-order process involving two electrons, other processes can also give rise to overbias emission. These include (1) blackbody radiation of hot carriers, in which the effective temperature is related to the bias voltage or the input power;^18−20,38,39^ (2) recombination of out-of-equilibrium carriers,^21^ in which electrons and holes in the high-energy tail of the Fermi–Dirac distribution tunnel into the TMD to form excitons; (3) second-order nonlinear optical processes, in which photons generated by IET^40,41^ excite excitons in the TMD; and (4) second-order energy transfer, in which the energy from pairs of coherently tunneling electrons^13−15^ is forming excitons in the TMD.

To exclude the first two processes, we fabricate yet another single-barrier MoSe_2_ LED and measure its EL at cryogenic temperature (∼10 K) (see the Supporting Information, Section VI). To rule out the thermal origin for the observed overbias emission, we use the following blackbody radiation model for the radiated power:^18−20^
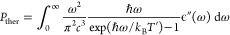
1where *c* is the speed of light, ω the photon angular frequency, *k*_B_ the Boltzmann constant, *T*′ the effective hot carrier temperature, and ϵ″ the emissivity of the TMD exciton, which can be derived from the refractive index.^42^ For resistive heating we obtain the linear dependence^19,20^
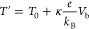
2where *T*_0_ is the lattice temperature and κ is a temperature-independent dimensionless constant that can be derived from experimental data at room temperature. With this κ, eq 1 predicts that the radiated power in the spectral region of the exciton should decrease by roughly 9 orders of magnitude when *T*_0_ is reduced from 300 to 10 K. However, our measurements show only a decrease of less than 2 orders of magnitude. This huge discrepancy between model and measurement indicates that blackbody radiation is not the source of the observed overbias emission. The same is true for the second scenario, the recombination of out-of-equilibrium carriers, because our measurements reveal that the dependence of the radiated power on bias voltage is unaffected by the lattice temperature (see the Supporting Information, Section VI, for analysis details).

The third scenario involves two steps, namely photon emission by IET^40,41^ and a subsequent nonlinear optical process. Comparing the photon emission efficiencies of the IET and the observed overbias emission, we require a nonlinear optical process with unit efficiency to explain our measurements. Therefore, it is safe to discard the third scenario as an explanation for our observation.

We are left with the fourth scenario, illustrated in Figure 4a. In this scenario, excitons are generated by the action of two electrons. This process is supported by two recent observations. First, it has been demonstrated that excitons can be efficiently excited by tunneling electrons via nonradiative energy transfer.^9^ Second, it has been reported that multielectron coherent tunneling can generate overbias emission in plasmonic tunnel junctions.^13−15,22,23^ Therefore, we identify the multielectron IET as the most likely mechanism responsible for the observed overbias emission.

**Figure 4:**
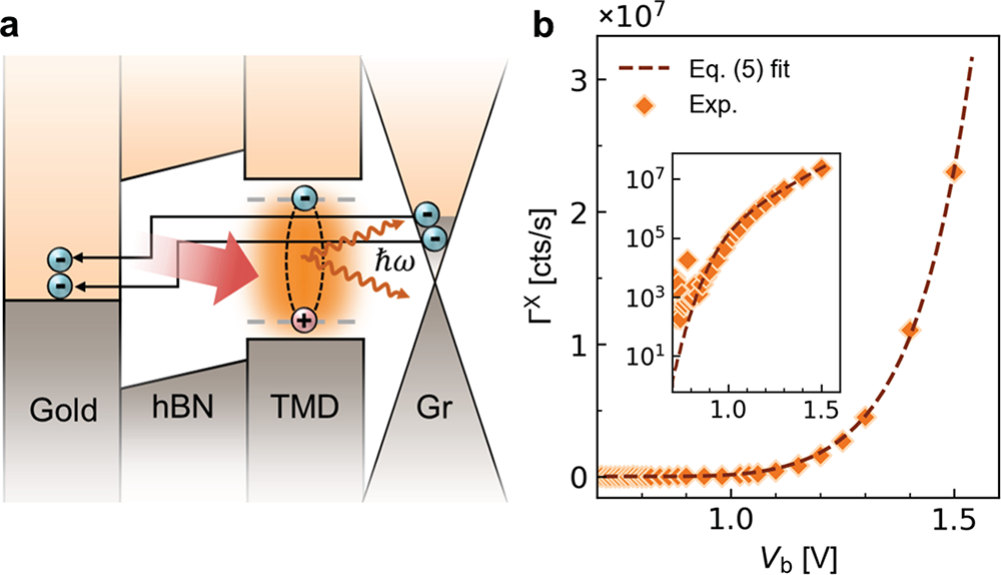
(a) Energy transfer based on two-electron coherent tunneling. A pair of electrons tunnel inelastically, and their combined energy generates excitons in the TMD. (b) Exciton EL intensity (Γ^X^) of a single-barrier WSe_2_ LED as a function of bias voltage *V*_b_. The inset shows the data on a semilogarithmic scale. The data points correspond to the integrated EL photon count rate in the spectral range from 1.4 to 1.8 eV. The dashed curve is the fitting result of eq 5.

In plasmonic tunnel junctions, overbias light emission based on two-electron IET depends on the interplay between higher-order quantum noise and the local density of optical states (LDOS).^13,14^ Here we adopt this theory to form a TMD-coupled tunnel junction. The nonsymmetrized power spectral density of the fluctuating tunnel current reads as^43,44^
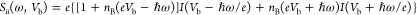
3where *I*(*V*_b_) is the bias-dependent tunnel current and  is the Bose–Einstein distribution at temperature *T*. We are concerned with the absorption of electromagnetic energy generated by the fluctuating tunneling current, which is described by the positive frequency part of *S*_*ii*_.^45^

The absorption depends on the local environment of the tunnel junction and is mathematically described by the LDOS (ρ) and the system’s Green’s function.^46^ For frequencies that correspond to the TMD exciton energy, the absorption is dominated by the LDOS of the TMD (ρ_TMD_). In a two-electron process, the locally absorbed energy is no longer linearly dependent on *S*_*ii*_. In analogy to previous studies,^15,22,23^ the two-electron energy absorption rate γ_2e_ can be represented as
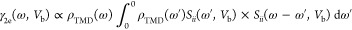
4where ρ_TMD_ is calculated by following ref (41). Equation 4 describes a two-electron tunneling process in which the energy of two electrons is absorbed by the TMD to generate an exciton (Figure 4a). Because excitons can only be generated by energies larger than the exciton energy (*ℏ*ω > *E*_X_), we can represent the exciton light emission intensity Γ^X^ as
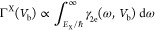
5As can be seen in Figure 4b, the exciton EL intensity increases exponentially with increasing *V*_b_, and the calculated Γ^X^(*V*_b_) according to eq 5 agrees well with the experimental results (see the Supporting Information, Section VIII). This agreement supports our interpretation that the overbias emission in our TMD-based LEDs results from two-electron tunneling, followed by energy transfer.

In summary, we investigated exciton light emission for potentials lower than the optical bandgap energy in TMD-based tunneling LEDs. We are able to measure exciton emission for bias potentials of only half the optical bandgap energy. We explain this overbiased emission by a second-order energy transfer process. Our work contributes to the understanding of light emission from vdW tunnel junctions and to the development of energy-efficient LEDs based on 2D materials.

## Supporting Information Available

Descriptions of sample fabrication, measurements methods, efficiency calculations of the LED, spectra processing and fitting methods, EL spectra acquired under the negative bias conditions of the device depicted in Figure 2, measurements and analysis of overbias emission at cryogenic environments, and the model of energy transfer based on the multielectron tunneling process (PDF)
