# Supplementary material for: Overbias Photon Emission from Light-Emitting Devices Based on Monolayer Transition Metal Dichalcogenides
Source: Nano Lett. 2023 Dec 4;23(23):10908–13. doi: 10.1021/acs.nanolett.3c03155 (PMC10722526; doi:10.1021/acs.nanolett.3c03155)
Supplement: Supplementary file 1 — nl3c03155_si_001.pdf [file nl3c03155_si_001.pdf]

Supporting Information: Overbias photon emission from light-emitting devices based on monolayer transition metal dichalcogenides

Shengyu Shan,[1,*] Jing Huang,[1,*] Sotirios Papadopoulos,[1] Ronja Khelifa,[1]
Takashi Taniguchi,[2] Kenji Watanabe,[3] Lujun Wang,[1] and Lukas Novotny[1,†]
[1]*Photonics Laboratory, ETH Zürich, 8093 Zürich, Switzerland*
[2]*International Center for Materials Nanoarchitectonics, National Institute for Materials Science, 1-1 Namiki, Tsukuba 305-0044, Japan*
[3]*Research Center for Functional Materials, National Institute for Materials Science, 1-1 Namiki, Tsukuba 305-0044, Japan*
(Dated: November 23, 2023)

I. SAMPLE FABRICATION

Monolayer graphene (Gr), monolayer transitional metal dichalcogenide (TMD), and multilayer hexagonal-Boron Nitride (hBN) flakes were mechanically exfoliated on top of SiO_2/Si substrate. Monolayer Gr and TMD were identified by optical contrast, while the thickness of hBN flakes were determined by atomic force microscope. The van der Waals (vdW) tunneling devices were assembled by a curved polydimethylsiloxane (PDMS) stamp covered with a polycarbonate (PC) film in a glovebox filled with Argon (Ar) gas [1]. A top hBN was picked up as the first layer to encapsulate the whole device. Different crystal flakes were picked up sequentially according to the design, and then the whole stack was transferred to a glass coverslip (or SiO_2/Si chip) with gold markers or electrodes. After the transfer, the PC film was dissolved in chloroform overnight. For the double-barrier LED, electrical contacts were made through Gr edge contacts by following Refs. [2, 3]. For the single-barrier LEDs, the Gr electrodes were placed directly on top of pre-patterned gold electrodes.

II. ELECTROLUMINESCENCE MEASUREMENT

At room temperature (setup-1)

The room temperature (RT) electroluminescence (EL) was measured on setup-1. The sample was placed on a customized inverted Nikon TE300 microscope equipped with a piezo stage. We used a Keithley 2602B source meter unit to apply voltage to the sample and the emitted light was collected with an objective. EL of the double-barrier LED was collected from the glass side with an oil immersion objective (Nikon, 100x, NA 1.4), while that of the single-barrier LEDs was collected from the air side with an air objective (Nikon, 100x, NA 0.9) due to the opaque bottom gold electrode. All spectra were recorded with a Princeton Instruments Acton SpectraPro 300i spectrometer. The light emission patterns were recorded by an Electron-multiplying charge-coupled device (EMCCD, Andor iXon Ultra).

At cryogenic temperature (setup-2)

The cryogenic temperature EL of the single-barrier $MoSe_2$ LED was measured on a different setup (setup-2) equipped with a cryostat (AttoDry 800). The sample was mounted on a cooling stage, facing up, and the EL was collected by an air objective (Thorlabs 350330-B, NA 0.68) from the top inside the same cooling chamber. After the objective, the light traveled through the optical window, and the spectra were recorded by a fiber-coupled spectrometer (Princeton Instruments Acton SP2500). We measured this device at RT on both setups for detection efficiency calibration. Thereby we can calibrate the EL intensity at cryogenic temperature (measured at setup-2) and compare it to that at RT (measured at setup-1). The bias voltage was applied by Keithley 2400 Sourcemeter. The tunneling LED was connected in series with a large protective resistor. The corresponding bias on the tunneling junction was assigned according to the current and another I-V characteristic measurement at cryogenic temperature. This I-V measurement

* These two authors contributed equally
† lnovotny@ethz.ch

was performed using a variable temperature probe station (Lake Shore Cryogenics, Model CRX-6.5K). The current was measured using a Femto DDPCA-300 current amplifier. An ADwin Pro II DAC was used to apply the bias voltage and read the output voltage of the current amplifier.

III. EFFICIENCY CALCULATION AND SPECTRA PROCESSING OF THE LEDS

The external quantum efficiency η_{EQE} of the LED, shown in Fig. 1d in the main text, is calculated as the number of emitted photons per second divided by the number of tunneling electrons per second:

$$\eta_{\mathrm{EQE}} = \frac{\text{\# Emitted photon per second}}{\text{\# Tunneling electron per second}} = \frac{\mathrm{sum}(\frac{\textit{raw spectra - background}}{t_{\mathrm{int}} \cdot \eta_{\mathrm{transmission}}})}{I/e}, \tag{S1}$$

where the I is the tunneling current, e is the elementary charge, *raw spectra* are the EL spectra as measured and the *background* is measured under the same condition with zero bias. t_{int} is the integration time of the spectra, and $\eta_{\mathrm{transmission}}$ represents the system transmission function of the setup-1, which is a combined transmission function of the detection path and the spectrometer data conversion. Its spectral dependence, i.e. the normalized transmission function, is calibrated by a Halogen light source (Ocean Optics, HL-2000-CAL) with known spectra. The absolute amplitude of the transmission function, related to the detection loss, is calibrated by measuring the reflection of a 532 nm continuous-wave laser on a gold surface with known laser incident power and sample reflectivity.

All EL spectra obtained at room temperature, presented in [cts/(s · eV)], represent the photon count rate per electronvolt (eV). Those spectra are calculated using the following expression:

$$\mathrm{EL}\ [\mathrm{cts}/(\mathrm{s}\cdot\mathrm{eV})] = \frac{\frac{\textit{raw spectra - background}}{t_{\mathrm{int}} \cdot \eta_{\mathrm{transmission}}}}{\Delta E\ [\mathrm{eV}]}, \tag{S2}$$

where ΔE [eV] denotes the energy difference between two adjacent pixels (or the corresponding wavelength of those pixels) of the spectrometer camera. In the end, the area of the spectra corresponds to the photon count rate entering the collection pathway.

However, *background* can vary slightly in each measurement. As a result, subtracting the *background* from the *raw data* introduces additional residual noise. Such residual noise typically manifests as white noise distributed across the measured wavelength range. Nevertheless, the unit conversion involving "ΔE [eV]" and the system transmission function $\eta_{\mathrm{transmission}}$ can distort this noise. These distortions become particularly pronounced when the signal-to-noise ratio is low. As shown in the main text (Fig. 1c, 2b, 2d, and 3a), the background noise consistently displays a slow decaying profile in the presented energy range.

The spectra shown in Fig. S3b, and c are corrected by a normalized transmission function $\eta_{\mathrm{transmission}}$ to obtain the correct shape of spectra. Fig. S3d, and e are calibrated by the collection efficiency of two setups.

IV. PSEUDO-VOIGT FUNCTION FITTING OF THE EL SPECTRA OF A MOSE$_2$-BASED SINGLE-BARRIER LED

Here we give several examples of pseudo-Voigt (PSV) function fitting of EL spectra, (taken from Fig. 3a in the main text), for a $MoSe_2$-based single-barrier LED. As shown in Fig. S1a, at low bias voltage (V_b = 0.76 V), the EL can only be fitted by a single PSV function with peak located at ∼1.56 eV (1s state of A-exciton [4]). As we increase the bias (V_b = 0.83 V), a second PSV function is included in order to obtain a better fitting result. This second PSV function has a peak centering at ∼1.66 eV (see Fig. S1b), which is attributed to the light emission from excited states of A-exciton according to its offset to A_{1s} feature [4]. At high bias voltage (V_b = 0.90 V), a third PSV function is added with peak centering at ∼1.74 eV, which is attributed to B-exciton [5], as plotted in Fig. S1c. For comparison, the fitting from another device is also shown in Fig. S1d, which shows similar features.

V. ELECTROLUMINESCENCE SPECTRA OF WSE$_2$-BASED SINGLE-BARRIER LED FOR DIFFERENT BIAS POLARITIES

In this work, we have observed devices with opposite asymmetric behaviors. i.e. the device in Fig. 2 (the same device as Fig. S2) is brighter at positive biases while the device shown in Fig. S3 is brighter at negative biases. We

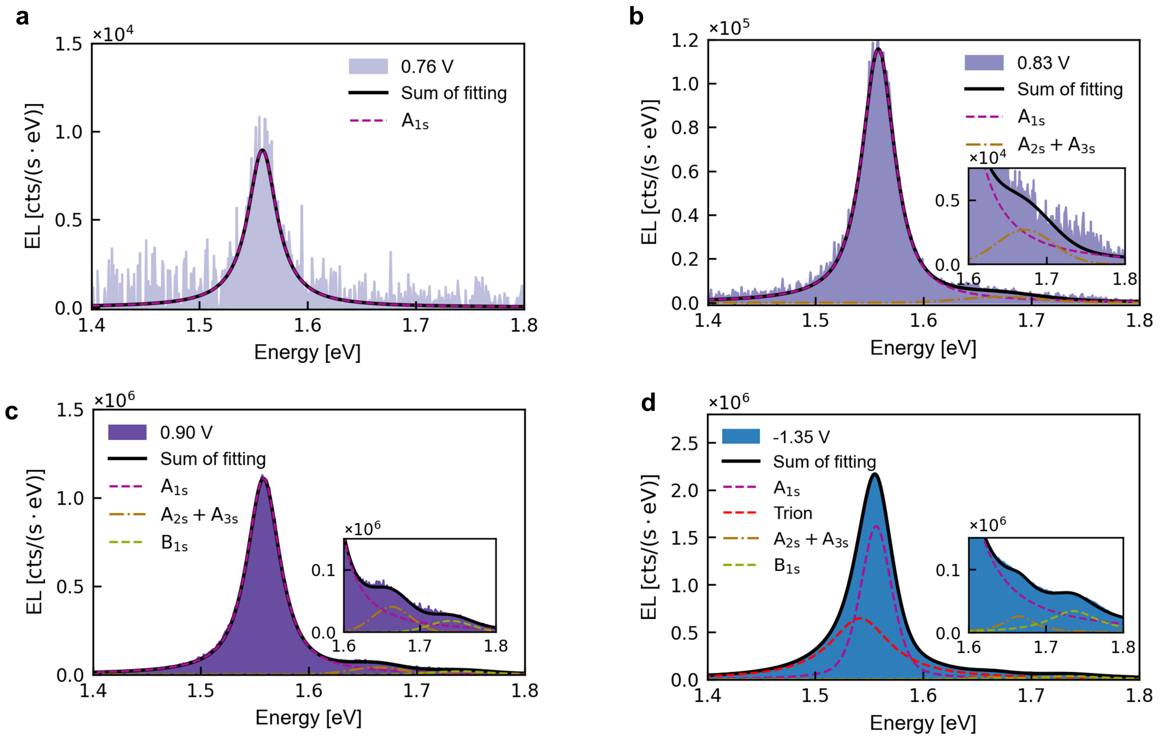

FIG. S1. **a**, **b** and **c**, EL spectra of a $MoSe_2$-based single-barrier LED under different biases and the corresponding PSV function fitting. These spectra were taken from Fig. 3a in the main text. In **c**, we fix the position of B exciton to 1.74 eV in the fitting due to the low signal-to-noise ratio. **d**, EL spectrum of another $MoSe_2$-based single-barrier LED and the corresponding fitting. This spectrum was taken from Fig. S3c. The A_{1s} is fitted with PSV function while all the other three features are fitted with the Lorentzian functions. The insets of **b**, **c**, **d** provide magnified views of part of the spectra.

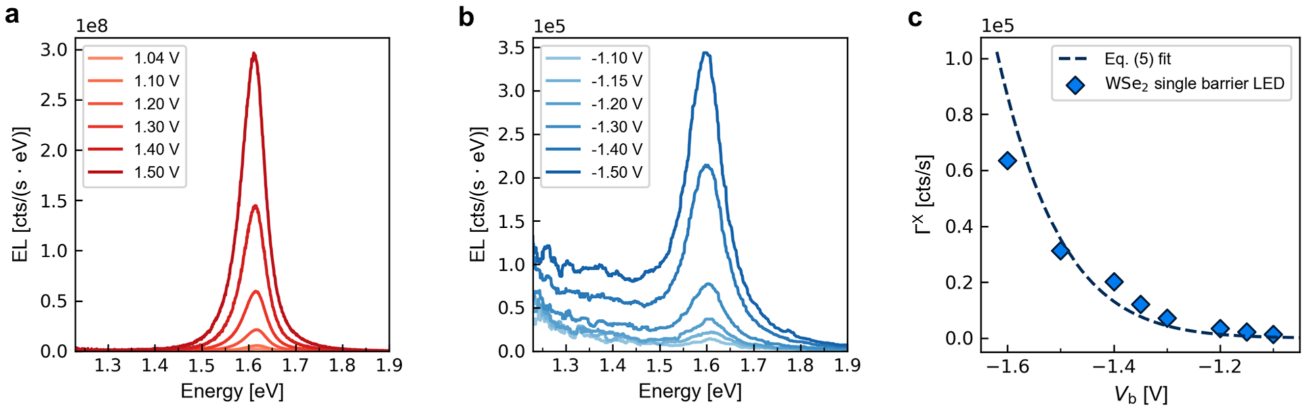

FIG. S2. **a** and **b**, EL spectra of WSe_2-based single-barrier LED (the same device shown in Fig. 2, main text) for several positive and negative biases, respectively. For negative bias, the A_{1s} exciton peak is around 1.60 eV, which is red-shift compared to positive bias (1.62 eV). **c**, Exciton EL intensity (Γ^X) of a single-barrier WSe_2 LED as a function of negative bias voltage V_b.

believe such asymmetry in overbias emission is mainly attributed to the differences among individual devices, rather than the nature of the energy transfer process.

We assume that one key factor determining the EL efficiency of a certain polarity is the effective doping of the TMD layer. This is given by the initial doping of the TMD plus the electrostatic gating. However, the bias voltage and effective doping level can not be controlled individually and we therefore observe brighter emission for a TMD

layer with less effective doping.

Additionally, we notice that for the brighter side (Fig. S2a, Fig. S3c), the exciton peak tends to have less redshift and narrower linewidth compared to the other side (Fig. S2b, Fig. S3b). Since the red-shift of A_{1s} exciton arises from the screening effect from Gr [4], more red-shift indicates a higher doping level of Gr. Meanwhile, the Gr-filtering effect depends on the doping level of Gr. A higher doping level means less effective filtering and the A_{1s} exciton is more broadened.

VI. CRYOGENIC MEASUREMENT AND ANALYSIS

A. Measurement results of a single-barrier $MoSe_2$ LED

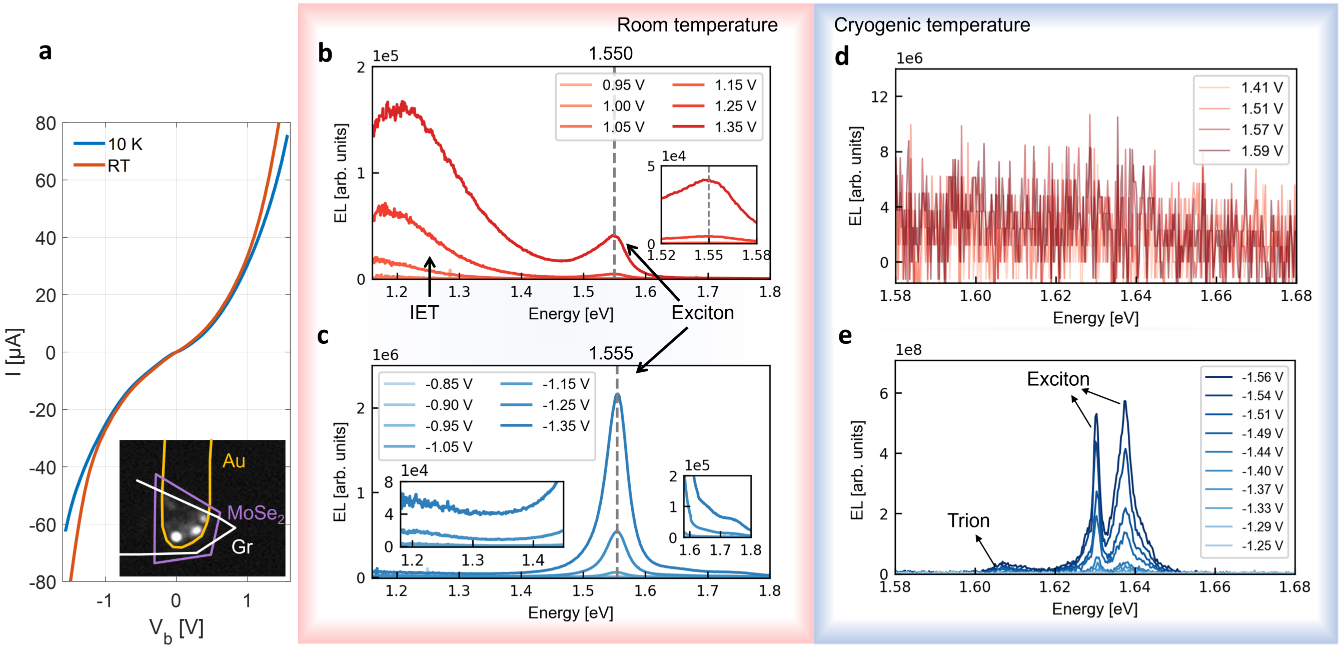

FIG. S3. Measurements of a single barrier $MoSe_2$ LED at room and cryogenic temperature. **a**, I-V characteristic at RT and ∼10 K. The inset is a sketch of the device overlaid with an EL picture recorded by EMCCD at RT. **b**, and **c**, Bias-dependent EL spectra for positive and negative polarities at RT. Spectra have been corrected by a normalized transmission function of setup-1. **d**, and **e**, Bias-dependent EL spectra for positive and negative polarities at cryogenic temperature (∼10 K).

One single-barrier $MoSe_2$ LED, was characterized at room and cryogenic temperatures to exclude the blackbody radiation of hot carriers and the contribution of non-thermal equilibrium band-edge carriers. Figure. S3a shows the I-V curve at RT and ∼10 K. The conductance decreases slightly when the junction cools down [6]. We define the Gr electrode as ground and gold as the anode (same as Fig. 2a, main text). Figures. S3b and c show the EL spectra at both polarities. In these two sets of spectra, we assign the broadband feature to the photon-assisted inelastic electron tunneling (IET) process [7] and the feature near 1.56 eV to exciton [4]. We observe the overbias exciton light emission at both polarities but with different intensities. We can not detect exciton light emission for even lower biases because of the thicker tunneling barrier (∼4 layers) compared with the LED presented in Fig. 3 of the main text (∼2 layers). A thicker barrier reduces the tunneling current density, as well as the exciton generation rate.

Figures S3d and e show the EL spectra when the device is cooled to near 10 K. For the positive bias (Fig. S3d), the exciton signal is too weak to be distinguished. This is likely caused by the lower exciton emission rate at positive bias when compared to negative bias (as seen in Figs. S3b and c) and the lower detection efficiency of setup-2. For the negative bias (Fig. S3e), the exciton features are clear. We assign the two main peaks to excitons and a small feature near 1.61 eV to trion according to the energy difference. The presence of multiple exciton features with comparable intensities could be attributed to the inhomogeneity of the tunnel junction (as can be seen in Fig. S3a inset). The trion signal may come from the region where $MoSe_2$ is decoupled to Gr [8]. The exciton EL intensity shown in Fig. S4a is calculated for the negative biases, i.e. spectra in Fig. S3c and e.

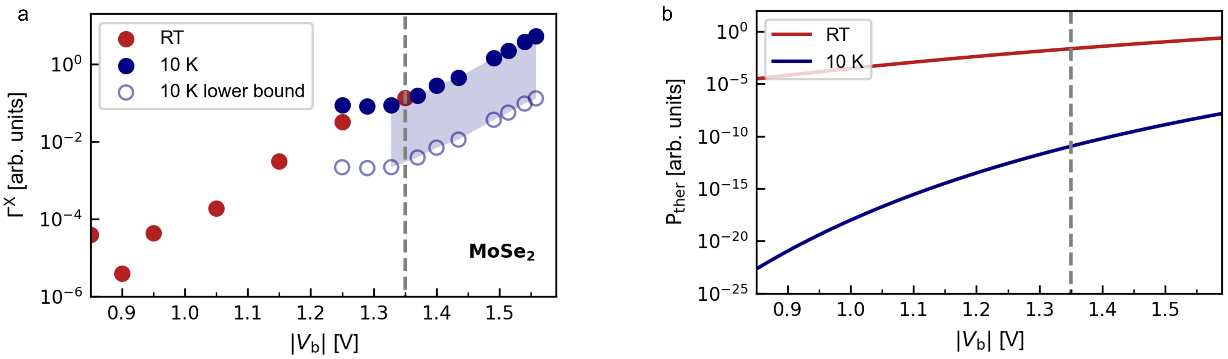

FIG. S4. **a**, Exciton EL intensity Γ^{X} at RT (red) and at 10 K (blue) as a function of bias voltage in log scale. Γ^{X} at RT and 10 K is evaluated by spectral integrating photon count rate from 1.4 eV to 1.8 eV and 1.61 eV to 1.65 eV respectively. Data at RT and at 10 K are measured in different setups (that result in the different noise floors) and calibrated afterward. Γ^{X} at 1.35 V almost remains the same after cooling. The unfilled blue circles come from a different calibration, which represents the lower bound of the photon count rate. **b**, Model of blackbody radiation at RT (red) and at 10 K (blue). The total radiated power shows a decrease of 9 orders of magnitude at 1.35 V.

B. Analysis of blackbody radiation model

The blackbody radiation spectrum of a thermal emitter can be described as [9–11]

$$P_{\mathrm{ther}}(\omega) = \int_0^\infty \frac{\omega^2}{\pi^2 c^3} \frac{\hbar\omega}{e^{\hbar\omega/k_{\mathrm{B}}T'} - 1} \epsilon''(\omega) d\omega, \tag{S3}$$

where c is the speed of light, and k_{B} is the Boltzmann constant, T' represents the effective temperature of the hot carriers, and ϵ'' accounts for the emissivity of the TMD exciton. Since the overbias emission is mainly from excitons, we extract $\epsilon''(\omega)$ near A-exciton energy by fitting the refractive index of $MoSe_2$ in Ref. [12] with a Lorentzian function. For resistive heating we assume T' depends linearly on the input voltage [10, 11]:

$$T' = T_0 + \kappa \frac{e}{k_{\mathrm{B}}} V_{\mathrm{b}}, \tag{S4}$$

where T_0 is the lattice temperature, e is the elementary charge, and κ is a temperature-independent dimensionless constant. If the source of the overbias emission would be blackbody radiation, then by fitting the RT bias-dependent exciton intensity Γ^{X} (red data points in Fig. S4a) with Eq. (S3), we can estimate $\kappa \simeq 0.023$. With this value of κ, Eq. (S3) gives us the results plotted in Fig. S4b, where the radiation power at 1.35 V drops by 9 orders of magnitude when T_0 decreases from 300 K to 10 K. However, in our measurement, the photon count rate almost remains the same (up to 2 orders of magnitude decrease according to another set of calibration, shown in unfilled circles), as shown in Fig. S4a. For comparison, the blackbody radiation model shows 2 orders of magnitude decrease when T_0 drops from 300 K to 210 K. Such a huge discrepancy between the model prediction and experimental results shows that the blackbody radiation model is not applicable to our devices. We can therefore clearly rule out the blackbody radiation mechanism. We note that we only consider the frequency range near the exciton energy at corresponding temperatures and assume the overall photon count rate Γ^{X} is proportional to the exciton radiation power P_{ther} in this small spectral range.

C. Analysis of non-thermal-equilibrium band-edge carriers

If the exciton generation is dominated by the high energy tail in Fermi-Dirac distribution [13], we assume the exciton EL intensity has the following expression [13]:

$$\Gamma^{\mathrm{X}} \propto \int_{E_{\mathrm{BG}}}^\infty \frac{1}{1 + \exp[(E - |eV_b|)/k_B T']} N_j(E) dE, \tag{S5}$$

where T' is the effective temperature, E_{BG} is the optical bandgap energy, which is the same as A-exciton energy, and N_j is the joint density of states. If we consider $E_{\mathrm{BG}} - |eV_b| \gg k_B T'$, assume that only the carriers near the band

edge dominate the exciton generation, and approximate Fermi-Dirac distribution to Maxwell-Boltzmann distribution, then in log scale, we have:

$$\log_{10}(\Gamma^{\mathrm{X}}) \sim \frac{1}{\ln(10)k_B T'} V_{\mathrm{b}} + C, \tag{S6}$$

where C is a constant, and the slope between $\log_{10}(\Gamma^{\mathrm{X}})$ and V_{b} is near $\sim \frac{1}{\ln(10)k_B T'}$. Cooling down the environment should effectively cool down the electron, thus increasing the slope, which is not consistent with our observation. As shown in Fig. S4a, the Γ^{X} dependence on V_{b} (in semi-logarithmic scale) has similar slopes at both temperatures. Thus we rule out as well the charge injection from the high energy tail governed by Fermi-Dirac distribution as an interpretation for the observed overbias emission. We note here that we can not directly compare the observed onset voltage due to the different collection efficiency of the two setups and different exciton energy at different temperatures.

VII. COMMENTS REGARDING NON-UNIFORM LIGHT EMISSION PATTERN

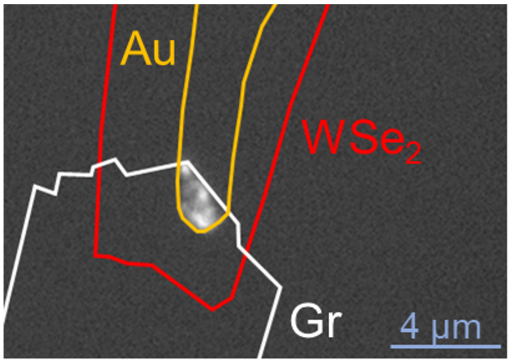

FIG. S5. Sketch of WSe_2-based single-barrier LED overlaid with its EL picture for $V_{\mathrm{b}} = 1.25\,\mathrm{V}$ at RT.

As shown in of Fig. S3a inset, the EL intensity of the $MoSe_2$-based LED has an inhomogeneous spatial distribution. Such non-uniformity is often associated with variations of the layer distance within the sample. Thickness variations in the tunneling tunnel barrier, particularly due to the presence of the roughness of the evaporated gold electrode, can result in non-uniform current injection. Apart from that, multiple factors listed below could also exacerbate the non-uniformity: 1) The separation variation between Gr and TMD, potentially arising from bubbles, can lead to different exciton quenching rates and changes to the dielectric environment, resulting in variations in intensity and spectral characteristics. This has been reported in Ref. [8]. 2) In our energy transfer model, the two-electron energy absorption rate γ_{2e} is highly sensitive to the separation variation between Gr and TMD. 3) Strain disorder can influence the exciton light emission patterns.

Such non-uniformity may raise concerns that overbias emission can only happen at specific spots due to unclear local properties, e.g. local increase in electron temperature. To exclude this concern and provide more insights, we present the EL picture of a WSe_2-based single-barrier LED for $V_{\mathrm{b}} = 1.25\,\mathrm{V}$ for comparison (Fig. S5). This emission pattern exhibits a more uniform EL intensity pattern with similar overbias emission properties. This indicates that overbias emission doesn't rely on non-uniform interfaces.

VIII. MODEL: ENERGY TRANSFERRED FROM MULTI-ELECTRON INELASTIC TUNNELING

A. Energy absorption from single- and multi-electron inelastic tunneling process

In a TMD-coupled vdW tunnel junction, TMD can absorb energy from IET events thanks to the ultimate proximity between different layers. Using the model developed in Ref. [14], the total energy absorption rate, $\gamma(\omega; V_{\mathrm{b}})$, due to IET can be expressed as a sum of single- and multi-electron tunneling processes:

$$\gamma(\omega, V_{\mathrm{b}}) = \gamma_{1e}(\omega, V_{\mathrm{b}}) + \gamma_{2e}(\omega, V_{\mathrm{b}}) + \gamma_{3e}(\omega, V_{\mathrm{b}})..., \tag{S7}$$

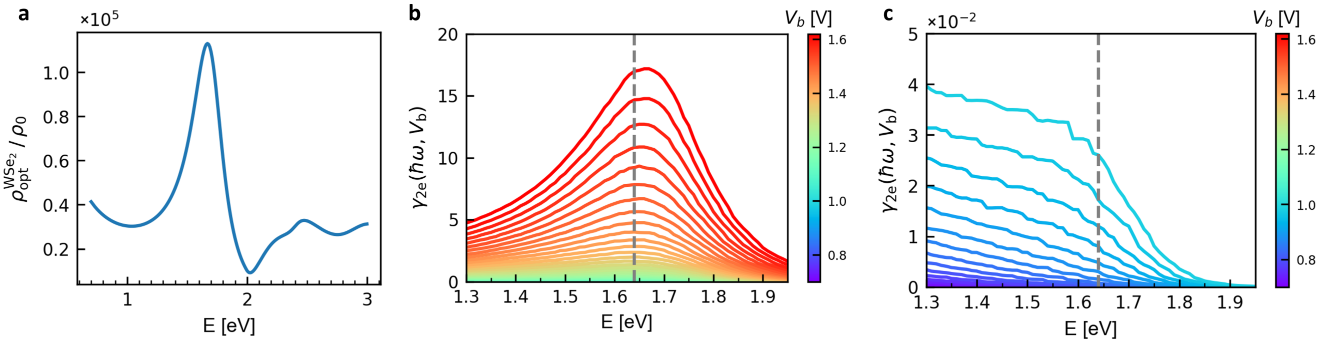

FIG. S6. **a**, Local density of optical state induced by WSe_2. **b** Calculated $\gamma_{2e}(\omega, V_\mathrm{b})$ for V_b ranging from 0.7 V to 1.64 V and **c**, 0.7 V to 1.00 V. The vertical dashed line denotes the threshold for exciton generation.

where ω corresponds to frequency near the TMD exciton resonance, V_b is the applied bias voltage. In the above expression, γ_{1e}, γ_{2e} and γ_{3e} represents the contribution from single-, two- and three-electron coherent tunnelings. For eV_b much smaller than the optical bandgap energy but still larger than half of it, energy transfer from single-electron tunneling can be neglected due to energy conservation. At the same time, the contribution from three-electron tunneling or even higher order processes is negligible compared to the two-electron process. Therefore, we are only interested in the two-electron energy absorption rate $\gamma_{2e}(\omega, V_\mathrm{b})$ which is given by Eq. (4) in the main text.

B. Local density of optical states ρ_TMD enhanced by TMD

In order to calculate the two-electron absorption spectrum $\gamma_{2e}(\omega, V_\mathrm{b})$, we need to know the power spectral density of the fluctuating tunnel current $S_{ii}(\omega, V_\mathrm{b})$ and the local density of optical states (LDOS) $\rho_\mathrm{TMD}(\omega)$ induced by TMD. While it is straightforward to estimate the former by Eq. (3) in the main text, the latter can be calculated through its relation to dissipated power P_dis of an electric dipole $\mathbf{p}$ in a layered structure, which reads as

$$P_\mathrm{dis} = \frac{\omega}{2}\mathrm{Im}\{\mathbf{p}^* \cdot \mathbf{E}(\mathbf{r}_0)\} = \frac{\omega}{2}p_\mathrm{z} \cdot \mathrm{Im}\{E_z(\mathbf{r}_0)\}, \tag{S8}$$

where $\mathbf{p} = p_\mathrm{z}\mathbf{z}$ with $\mathbf{z}$ corresponding to the unit vector of direction of current flow (perpendicular to the heterostructure), $\mathbf{E}(\mathbf{r}_0)$ is the electric field at the position of dipole $\mathbf{r}_0$, which is a superposition of the primary dipole field and the scattered field arising from the interaction with the environment. For a dipole source embedded in a layered structure, $\mathbf{E}(\mathbf{r}_0)$ can be calculated by following Ref. [7, 15]. The total optical density of state ρ is then calculated by using the following relation:

$$\frac{\rho}{\rho_0} = \frac{P_\mathrm{dis}}{P_0}, \tag{S9}$$

where ρ_0 and P_0 are known reference values, representing the density of optical states and the dissipated power of a radiating dipole in vacuum, respectively.

To extract the LDOS induced by monolayer TMD (e.g. WSe_2), we use the same approximation as in Ref. [16]. We simplify the system to an out-of-plane electric dipole near a monolayer WSe_2 with distance d, and assume $\rho_{\mathrm{WSe}_2} = \rho(d = 0.6\,\mathrm{nm}) - \rho(d \to \infty)$ for the single-barrier LED presented in main text Fig. 2. As shown in Fig. S6a, the calculated ρ_{WSe_2} as a function of photon energy shows a peak corresponding to the A-exciton energy of WSe_2. By inserting ρ_{WSe_2} into Eq. (4) in the main text, we obtain the bias-dependent two-electron energy absorption spectra, as shown in Fig. S6b and c. By inserting γ_{2e} into Eq. (5) in the main text, i.e. assuming the exciton EL intensity Γ^X is proportional to the integral of $\gamma_{2e}(\omega, V_\mathrm{b})$ in the energy range from WSe_2 exciton energy 1.64 eV (grey dashed line) to infinity, we obtain the fitting result in Fig. 4b, with the prefactor as the only fitting parameter.

[1] P. J. Zomer, M. H. Guimarães, J. C. Brant, N. Tombros, and B. J. Van Wees, Fast pick up technique for high quality heterostructures of bilayer graphene and hexagonal boron nitride, Appl. Phys. Lett. **105**, 013101 (2014).

[2] L. Wang, I. Meric, P. Y. Huang, Q. Gao, Y. Gao, H. Tran, T. Taniguchi, K. Watanabe, L. M. Campos, D. A. Muller, J. Guo, P. Kim, J. Hone, K. L. Shepard, and C. R. Dean, One-dimensional electrical contact to a two-dimensional material, Science **342**, 614 (2013).

[3] H. Overweg, H. Eggimann, X. Chen, S. Slizovskiy, M. Eich, R. Pisoni, Y. Lee, P. Rickhaus, K. Watanabe, T. Taniguchi, V. Fal'Ko, T. Ihn, and K. Ensslin, Electrostatically Induced Quantum Point Contacts in Bilayer Graphene, Nano Lett. **18**, 553 (2018).

[4] E. Lorchat, L. E. López, C. Robert, D. Lagarde, G. Froehlicher, T. Taniguchi, K. Watanabe, X. Marie, and S. Berciaud, Filtering the photoluminescence spectra of atomically thin semiconductors with graphene, Nat. Nanotechnol. **15**, 283 (2020).

[5] B. Han, C. Robert, E. Courtade, M. Manca, S. Shree, T. Amand, P. Renucci, T. Taniguchi, K. Watanabe, X. Marie, L. E. Golub, M. M. Glazov, and B. Urbaszek, Exciton states in monolayer $mose_2$ and $mote_2$ probed by upconversion spectroscopy, Phys. Rev. X **8**, 031073 (2018).

[6] F. Withers, O. Del Pozo-Zamudio, S. Schwarz, S. Dufferwiel, P. M. Walker, T. Godde, A. P. Rooney, A. Gholinia, C. R. Woods, P. Blake, S. J. Haigh, K. Watanabe, T. Taniguchi, I. L. Aleiner, A. K. Geim, V. I. Fal'Ko, A. I. Tartakovskii, and K. S. Novoselov, WSe_2 Light-Emitting Tunneling Transistors with Enhanced Brightness at Room Temperature, Nano Lett. **15**, 8223 (2015).

[7] M. Parzefall, Á. Szabó, T. Taniguchi, K. Watanabe, M. Luisier, and L. Novotny, Light from van der Waals quantum tunneling devices, Nat. Commun. **10**, 292 (2019).

[8] G. Froehlicher, E. Lorchat, and S. Berciaud, Charge Versus Energy Transfer in Atomically Thin Graphene-Transition Metal Dichalcogenide van der Waals Heterostructures, Phys. Rev. X. **8**, 11007 (2018).

[9] M. Buret, A. V. Uskov, J. Dellinger, N. Cazier, M. M. Mennemanteuil, J. Berthelot, I. V. Smetanin, I. E. Protsenko, G. Colas-Des-Francs, and A. Bouhelier, Spontaneous Hot-Electron Light Emission from Electron-Fed Optical Antennas, Nano Lett. **15**, 5811 (2015).

[10] L. Cui, Y. Zhu, M. Abbasi, A. Ahmadivand, B. Gerislioglu, P. Nordlander, and D. Natelson, Electrically Driven Hot-Carrier Generation and Above-Threshold Light Emission in Plasmonic Tunnel Junctions, Nano Lett. **20**, 6067 (2020).

[11] Y. Zhu, L. Cui, and D. Natelson, Hot-carrier enhanced light emission: The origin of above-threshold photons from electrically driven plasmonic tunnel junctions, J. Appl. Phys. **128**, 233105 (2020).

[12] H. L. Liu, T. Yang, J. H. Chen, H. W. Chen, H. Guo, R. Saito, M. Y. Li, and L. J. Li, Temperature-dependent optical constants of monolayer MoS_2, $MoSe_2$, WS_2, and WSe_2: spectroscopic ellipsometry and first-principles calculations, Sci. Rep. **10**, 15282 (2020).

[13] See Supplementary Information for: Y. Lian, D. Lan, S. Xing, B. Guo, Z. Ren, R. Lai, C. Zou, B. Zhao, R. H. Friend, and D. Di, Ultralow-voltage operation of light-emitting diodes, Nat. Commun. **13**, 3845 (2022).

[14] P.-J. Peters, F. Xu, K. Kaasbjerg, G. Rastelli, W. Belzig, and R. Berndt, Quantum coherent multielectron processes in an atomic scale contact, Phys. Rev. Lett. **119**, 066803 (2017).

[15] L. Novotny and B. Hecht, *Principles of nano-optics* (Cambridge university press, 2012).

[16] S. Papadopoulos, L. Wang, T. Taniguchi, K. Watanabe, and L. Novotny, Energy transfer from tunneling electrons to excitons (2022), arXiv:2209.11641 (accessed November 15th, 2023).
